# Prescribing and Monitoring of High-Dose Antipsychotic Therapy in Adults: Are We Getting HDAT Right?

**DOI:** 10.1192/bjo.2026.11666

**Published:** 2026-06-30

**Authors:** Rebecca McKnight, Emily Kiernan, Mohammad Umair Hafeez, Victoria Jones, Adeola Akinola

**Affiliations:** 1 Greater Manchester Mental Health NHS Foundation Trust, Manchester, United Kingdom; 2 Manchester Metropolitan University, Manchester, United Kingdom; 3 Pennine Care NHS Foundation Trust, Manchester, United Kingdom; 4 The University of Manchester, Manchester, United Kingdom

## Abstract

**Aims:**

High Dose Antipsychotic Therapy (HDAT) is defined as prescribing a single antipsychotic above the British National Formulary (BNF) maximum or a combinedpercentage dose of two or more antipsychotics exceeding this limit.HDAT is off-label and associated with increased morbidity and mortality, requiring documented clinical justification and risk–benefit discussion.This audit evaluated HDAT prevalence, adherence to Trust monitoring standards and reported side effects among adults prescribed antipsychotics in inpatient and community settings.

**Methods:**

A cross-sectional audit was conducted between May and August 2025 of adults aged 18–65 years receiving antipsychotic therapy.All patients in nine inpatient psychiatric wards and a random sample of patients from two Community Mental Health Teams and one Early Intervention in Psychosis service were audited.Prescription charts, clinic letters and medical records were reviewed to assess prescribing patterns, HDAT prevalence, compliance with Trust monitoring standards, patient demographics, psychiatric diagnosis, treatment duration, and medication side effects.

**Results:**

A total of 337 patients were audited (n=142 female, 42.1%; n=195 male, 57.9%), mean age 41 years. Most identified as White (76.9%), with 19.0% Black or Asian and 4.2% unknown ethnicity. The majority (72.7%) were prescribed at least one additional psychotropic medication and 18.1% reported side effects, predominantly extrapyramidal symptoms (52.5%), weight gain (24.6%), and sedation (23%).Thirteen patients (3.9%) met HDAT criteria, primarily in inpatient settings (n=11); mean age 43 years.Most were male (84.6%), White (92.3%) and diagnosed with schizophrenia (84.6%).Over half (69.2%) received two antipsychotics, whilst 30.7% were prescribed a single antipsychotic above BNF limits.HDAT monitoring was fully compliant in 38.5% of cases, partially in 23.0%, and incomplete in 30.8%.Fewer HDAT patients reported medication side effects (15.4%).In the overall, sample, 32.6% had received antipsychotic treatment for over 12 months, rising to 46.2%among those on HDAT.

**Conclusion:**

HDAT was infrequent, largely confined to inpatient settings and typically associated with antipsychotic polypharmacy and co-administered psychotropics.White men were more likely to receive HDAT, despite a broadly balanced gender distribution overall. Frequent side effects, relatively young cohort and known risks of HDAT highlights the importance of adherence to Trust monitoring standards, which was achieved in fewer than half of cases. The low rate of side effects among patients receiving HDAT likely reflects under-recognition or under-reporting rather than true tolerability, particularly in the context of polypharmacy and long-term treatment.These findings support the need for targeted clinician education and improved systems to promote safer HDAT practice.

